# Global research trends and hotspots in prognostic prediction models for pancreatic cancer: a bibliometric analysis

**DOI:** 10.3389/fonc.2025.1588735

**Published:** 2025-07-10

**Authors:** Siyuan Ouyang, Jing Zhang, Fuyao Liu, Qi Jiang, Wei Xing, Jie Chen, Jinggang Zhang

**Affiliations:** ^1^ Department of Radiology, The Third Affiliated Hospital of Soochow University, Changzhou, China; ^2^ Magnetic Resonance Research Collaboration Team, Siemens Healthineers Ltd., Shanghai, China

**Keywords:** pancreatic cancer, prediction model, prognosis, bibliometrics, trends

## Abstract

**Background:**

Pancreatic cancer is a highly aggressive malignancy of the digestive system, characterized by insidious onset and rapid progression. Most cases are diagnosed at advanced stages, complicating surgical resection and presenting significant challenges for clinical treatment. Recent advancements have emphasized individualized treatment strategies tailored to patients’ specific conditions. Consequently, accurate preoperative assessment is crucial, highlighting the urgent need to develop more reliable predictive models to guide personalized treatment plans.

**Methods:**

A systematic literature search was conducted using Web of Science Core Collection (WoSCC) database, covering publications from January 1, 1995, to October 25, 2024. A comprehensive bibliometric analysis was performed employing analytical tools such as VOSviewer, CiteSpace and Microsoft Excel.

**Results:**

This study includes 919 publications authored by 6716 researchers from 3727 institutions in 222 countries and regions. The articles were published in 301 journals, with 1,640 distinct keywords and 25,910 references. China led in publication volume, while the United States garnered the most citations. The top three research institutions in this field were Fudan University, Shanghai Jiao Tong University, and Sun Yat-sen University. Yu Xianjun from Fudan University emerged as the most prolific author with the highest citation count. *Frontiers in Oncology* had the highest publication volume, while the *Annals of Surgery* received the most citations. Medical imaging, biochemistry, immunology, bioinformatics, genetics, and interdisciplinary integrative research are the main research disciplines in the field of prognosis prediction for pancreatic cancer. The results of keyword co-occurrence and literature co-citation analysis revealed emerging hotspots and trends in this field, including CA19-9, CT, inflammation, machine learning, tumor microenvironment, radiomics, genes, nomograms, randomized controlled trials, long-term survival, and metastasis.

**Conclusion:**

This bibliometric analysis provides an overview of research conducted over the past three decades, offering insights into the current state of knowledge and outlining directions for future studies on prognosis prediction models for pancreatic cancer. Biochemical indicators have consistently emerged as key research focal points. The tumor microenvironment represents a currently popular research direction, while bioinformatics, medical imaging, and artificial intelligence are gaining traction as future trends in this field. In the future, prognostic models for pancreatic cancer require further refinement to ensure reliable guidance for therapeutic decision-making.

## 1 Introduction

Pancreatic cancer is a highly lethal malignancy of the digestive system with poor prognosis and persistently high mortality rates. According to the latest data from the American Cancer Society, the 5-year relative survival rate for pancreatic cancer remains alarmingly low at approximately 13%. In 2024, an estimated 51,750 deaths are expected from pancreatic cancer in the United States, making it the third-leading cause of cancer-related death in the country. Since the mid-1970s, the incidence of pancreatic cancer has increased by 1% annually (1), and it is projected that by 2030, pancreatic cancer will surpass breast cancer as the second-leading cause of cancer-related deaths (2). Cancer-related statistics in China also show a continuous upward trend in the incidence of pancreatic cancer from 2000 to 2018, with the mortality rate of pancreatic cancer in China is higher than that in the United States (3). Surgical resection is currently the only definitive treatment for pancreatic cancer, but only 10% to 20% of patients diagnosed with the disease are candidates for surgery (4). For patients with borderline resectable and metastatic disease, neoadjuvant therapy is often considered a treatment option (5, 6), though accurate risk stratification is critical to enhancing its clinical effectiveness. Therefore, due to the uncertainty of the prognosis of pancreatic cancer, there is a growing interest in prognostic prediction models for pancreatic cancer (7).

Prediction models are statistical tools designed to objectively, accurately, and reproducibly quantify the probability of an individual experiencing a specific event (such as survival or metastasis) under specific risk factors. Prognostic prediction models for pancreatic cancer can forecast the prognostic outcomes of different treatment regimens (such as neoadjuvant therapy or surgery) (8), thereby providing valuable data to support treatment selection and risk assessment. In recent years, with in-depth research and understanding of various prognostic factors, more reliable prognostic prediction models have been developed, but there is currently a lack of comprehensive evaluation of these models.

Bibliometrics involves the use of statistical data to analyze published information, such as books, journal articles, datasets, along with related metadata like abstracts, keywords, citation, in order to explore the relationships between these works (9). This method has become a crucial way for assessing the quality and impact of academic works. The objective of this study is to examine the research landscape of prognostic prediction models for pancreatic cancer through bibliometric analysis, providing direction and new ideas for future research in this field for researchers.

## 2 Methods

### 2.1 Search strategies

**Figure 1:**
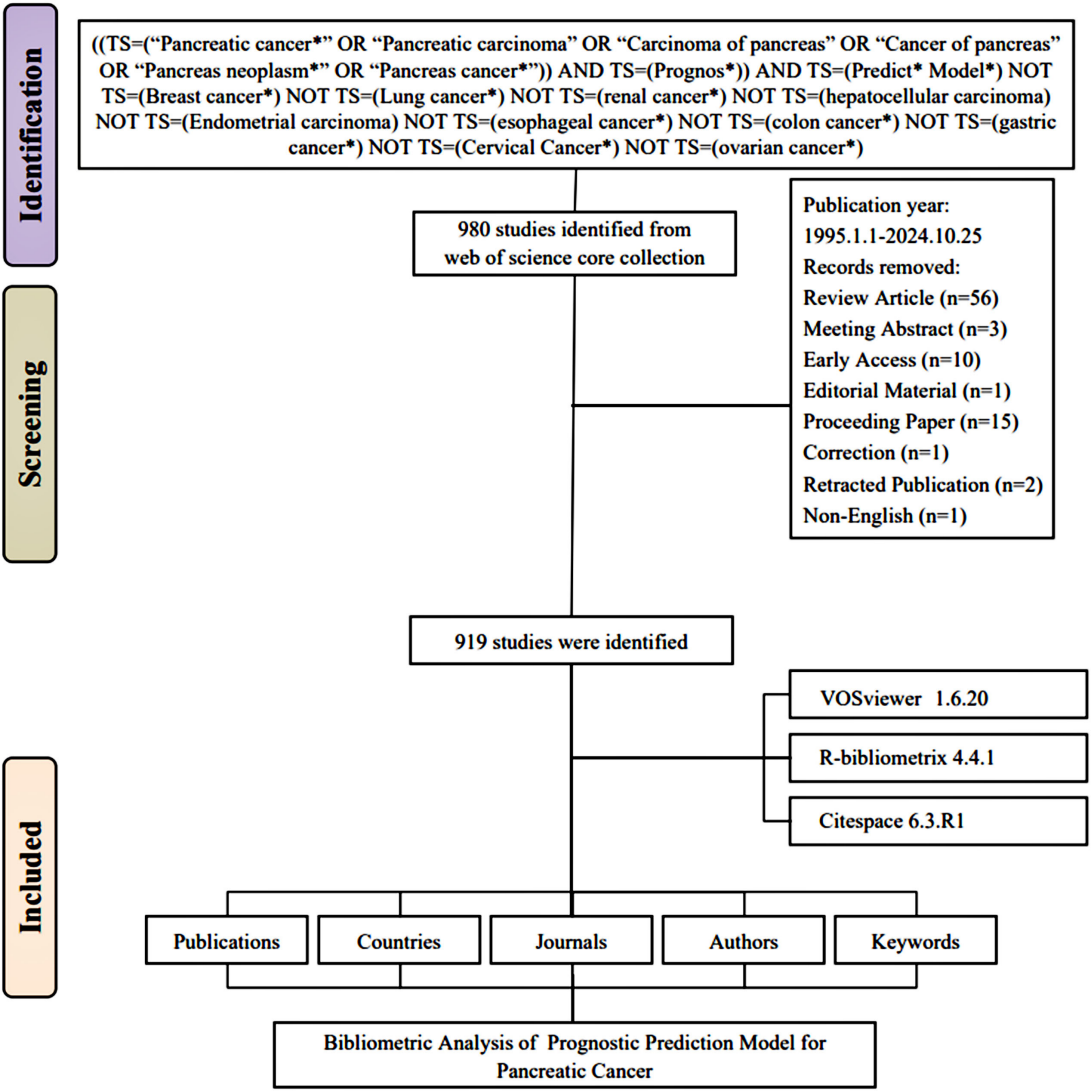
Flowchart of the literature screening process.

The Web of Science Core Collection (WoSCC) database was utilized to identify relevant publications. WoSCC c is widely recognized for indexing only high-quality journals, making it the most used database for bibliometric studies. The literature search covered a 30-years period, from January 1, 1995, to October 25, 2024. The search strategy referenced previous related research (10) and set the following search criteria: 1) “Pancreatic tumor*”,”Prognostic”, “Predict* Model” were used as the subject keywords for the search. 2) Considering that most prognostic prediction models for pancreatic cancer are experimental studies, only articles of the “Article” type were accepted. 60 other types of research were excluded.3) One non-English literature was excluded. 4) To reduce potential systematic bias resulting from database search result deviations, a comprehensive search and screening of publications were conducted, and the search query was further updated to exclude irrelevant literature. The final search query is as follows: ((TS=(“Pancreatic cancer*” OR “Pancreatic carcinoma” OR “Carcinoma of pancreas” OR “Cancer of pancreas” OR “Pancreas neoplasm*” OR “Pancreas cancer*”)) AND TS=(Prognos*)) AND TS=(Predict* Model*) NOT TS=(Breast cancer*) NOT TS=(Lung cancer*) NOT TS=(renal cancer*) NOT TS=(hepatocellular carcinoma) NOT TS=(Endometrial carcinoma) NOT TS=(esophageal cancer*) NOT TS=(colon cancer*) NOT TS=(gastric cancer*) NOT TS=(Cervical Cancer*) NOT TS=(ovarian cancer*). The specific flow chart was shown in **Figure 1**.

### 2.2 Data analysis

The included publications and cited references were exported as plain text for bibliometric analysis and visualization. Flowcharts were created using Figdraw (http://www.figdraw.com). Statistical analysis was performed using the bibliometrix package (v4.4.1) in R software (v4.4.1), while Microsoft Excel was employed for data aggregation, statistical table generation, and trend chart creation. Subsequently, VOSviewer (v1.6.20), CiteSpace (v6.3.R1), and Scimago Graphica (v1.0.35; https://www.graphica.app/) were utilized to analyze complex relationship networks among countries/regions, institutions, authors, journals, keywords, and references. In the network graphs, different clusters in the network graph are represented by different colors, with collaborations or co-citations indicated by connecting lines. The size of the circles represents the number of documents, references, or keywords, while the thickness of the connecting lines represents the strength of the links. To ensure the analytical accuracy, we also implemented several data cleaning measures, including merging nodes with similar semantics, correcting spelling errors, and unifying terms with different expressions. For example, Pancreatic Adenocarcinoma, Pancreatic Cancer, and Pancreatic Ductal Adenocarcinoma are considered different expressions of the same terminology, with nodes unified as Pancreatic Ductal Adenocarcinoma, while immune microenvironment and tumor microenvironment are considered nodes with similar semantics. In CiteSpace statistics, these nodes are merged into the same node: tumor microenvironment. Additionally, to gain a deeper understanding of the research on prognostic prediction models for pancreatic cancer, we conducted an analysis of the keywords from the retrieved literature. This included keyword co-occurrence analysis using VOSviewer and keyword burst detection with CiteSpace. Moreover, the quantity and quality of researchers’ academic achievements are evaluated using the H-index proposed by Hirsch (11). If a scientist has published n papers within a certain period, and h of these papers have been cited at least h times, with the citation counts of the other papers being ≤h, then the scientist’s H-index is h.

## 3 Results

### 3.1 Trends in the growth of publications

**Figure 2:**
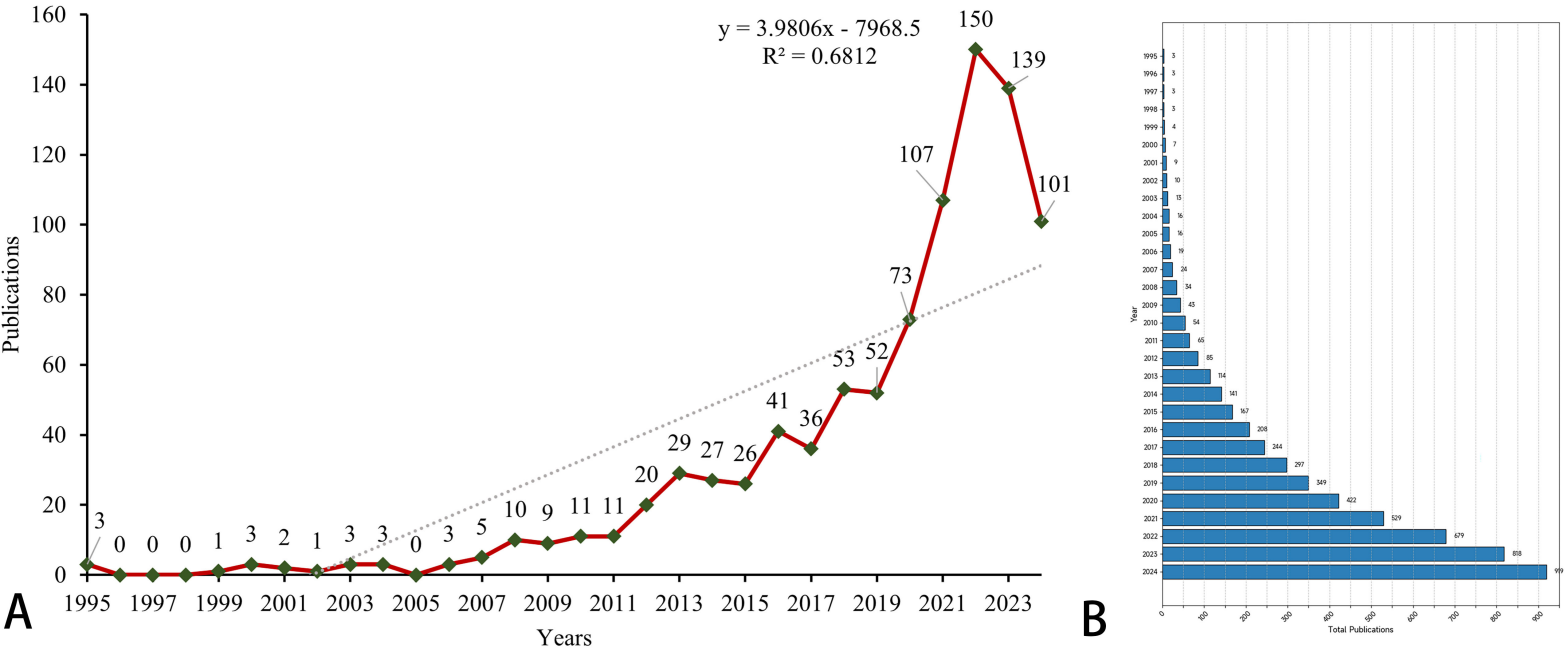
The global number of publications. **(A)** A line chart of the number of annual publications. The green dot represents the specific data of each year, and the red line represents the trend line of the data. The diagonal line in the figure represents the growth trend. **(B)** A clustered bar graph of the cumulative number of publications.

The overall growth trend in the annual number of publications on prognostic models for pancreatic cancer, as well as the distribution of publications by country, is illustrated in **Figure 2**. Between 1995 and 2024, the WoSCC core database documented a total of 919 publications on prognostic models for pancreatic cancer. Specifically, the evolution of this field can be delineated into four stages: 1) The Emerging Stage (1996–2011), where the volume of publications remained consistently low. 2) The Growing Stage (2012–2018), characterized by fluctuating but gradually increasing publication numbers. 3) The Rapid Expansion Stage (2019–2022), during which the number of publications surged. 4) The Stabilization Stage (2023-2024), where the publication volume remained consistently high. As shown in **Figure 2A**, the publication trend follows the linear equation y = 3.9806x - 7968.5 (x = year, y = publication count). Polynomial regression (R² = 0.6812) confirms a significant positive correlation between publication year and output volume (p*< 0.001), indicating continued high productivity. **Figure 2B** shows a clustered bar graph of the cumulative number of publications. the cumulative number of publications has increased rapidly.

### 3.2 Situation of countries/regions and institutions

**Figure 3:**
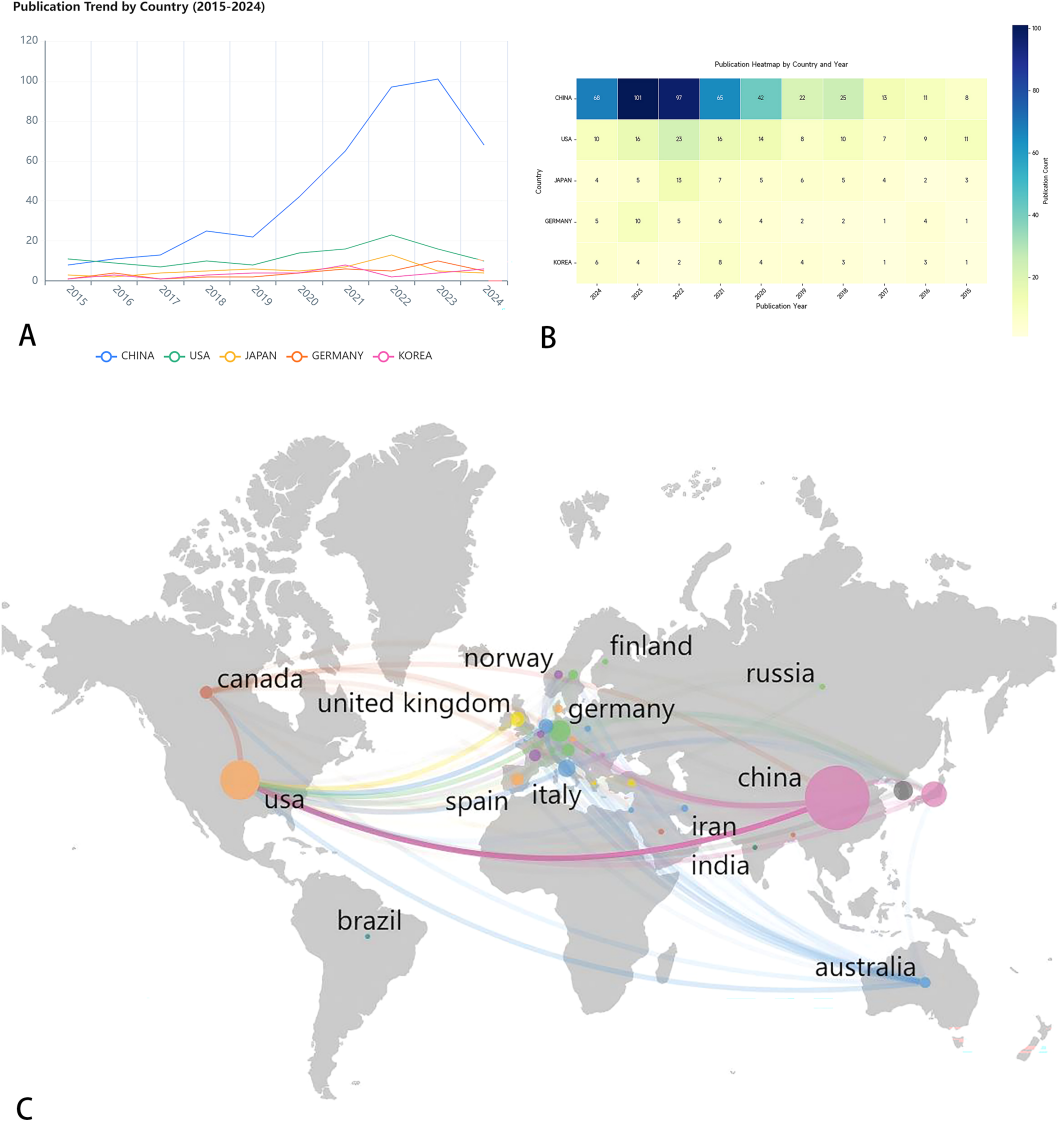
**(A)** Line chart of annual publication volume of the top five countries. This line chart shows the publishing volume trend of five countries (China, the United States, Japan, Germany and South Korea) from 2015 to 2024. **(B)** Annual publication heat map of the top five countries in terms of publication volume. Use different color shades to indicate the change in the number of publications per year. **(C)** Country/region collaboration map for prognostic prediction model for pancreatic cancer studies. Through the lines of different colors, it illustrates the academic exchanges between countries.

As shown in **Table 1**, research on pancreatic cancer prognostic models has been published across 222 countries/regions. The table ranks the top 10 by publication volume, with China leading (477 papers), followed by the United States (128 papers) and Japan (65 papers). In terms of citation counts, the USA ranks first (5,836 citations), followed by China (5,827 citations) and Japan (1,645 citations). Despite China has the highest publication volume, the average number of citations per paper (N=12.2) is considerably lower than that of the USA (N=45.6) and Japan (N=25.3), which may indicate that the academic impact of Chinese scholars’ articles is relatively low. **Figure 3A** displays the trend of literature publication in different countries from 2015 to 2024. As can be seen from the figure, the number of published literatures (blue line) in China has increased significantly after 2018. The United States (red line) and South Korea (green line) maintained a relatively stable number of publications. Other countries have published less in this field, and there is no obvious upward trend. **Figure 3B** presents an annual publication heatmap (2015–2024), where darker shades indicate higher publication counts per country/year. For example, China (dark blue) dominated publications in 2023. **Figure 3C** presents a network diagram illustrating inter-country collaboration, where larger nodes for China and the USA represent a relatively higher publication volume. The connecting lines between countries and their thickness reflect the degree of collaboration. As shown in the **Figure 3**, East Asia (including China, Japan, and South Korea), North America, and Europe are the three main collaboration clusters, with the thickest line between China and the USA indicating the closest collaboration.

**Table 1:** The top 10 countries/regions for publications.

Country	Articles	Total citations	Average citations
CHINA	477	5827	12.2
USA	128	5836	45.6
JAPAN	65	1645	25.3
KOREA	39	649	16.6
GERMANY	36	1339	37.2
ITALY	28	1010	36.1
NETHERLANDS	16	347	21.7
UNITED KINGDOM	14	400	28.6
AUSTRIA	13	846	65.1
SPAIN	13	345	26.5

**Figure 4:**
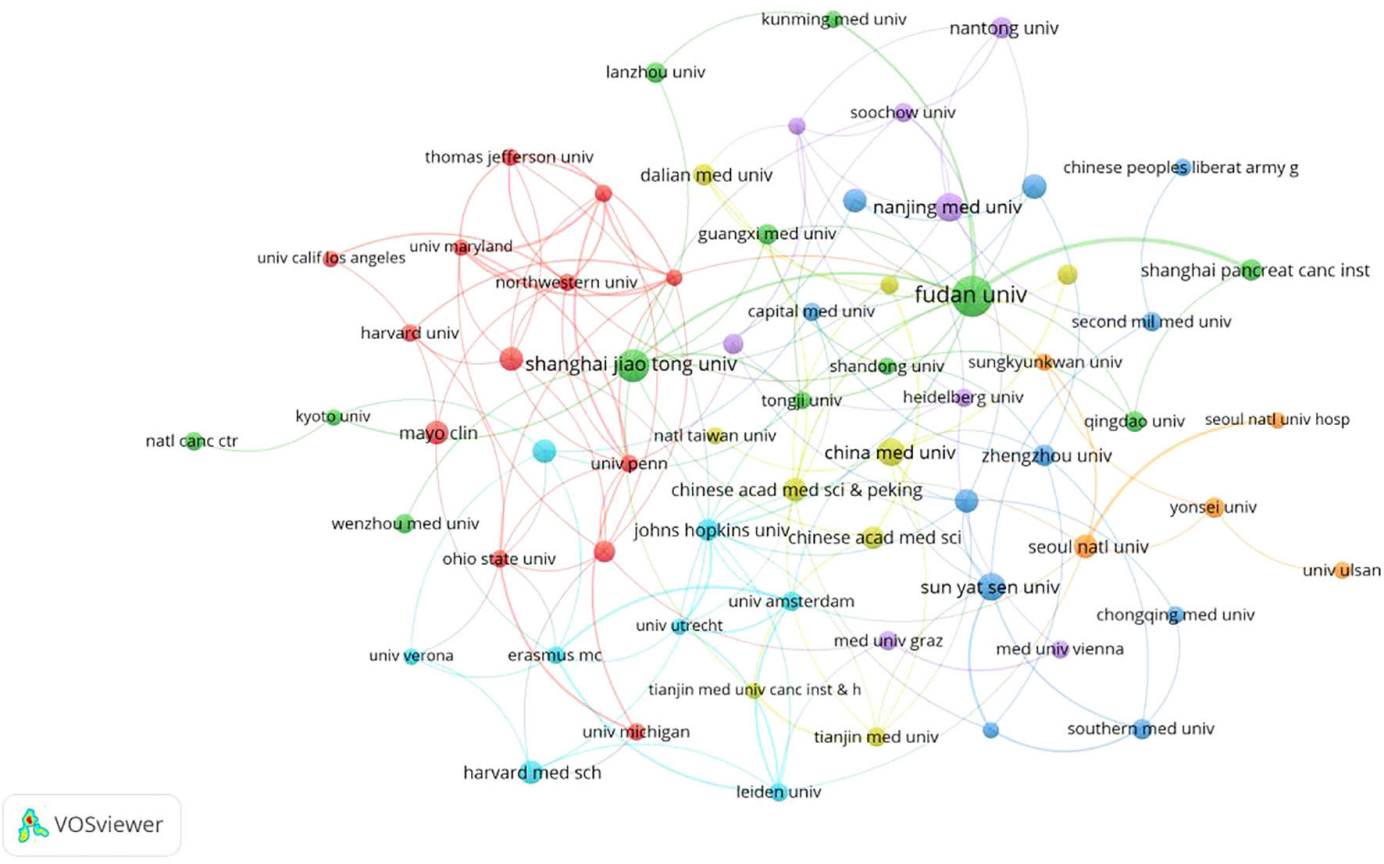
Institutional network map for prognostic prediction model for pancreatic cancer.

The situation of institutions is summarized in **Table 2** and **Figure 4**. **A** total of 301 institutions globally have contributed to contributed to pancreatic cancer prognostic model research. **Table 2** lists the top five institutions by publication volume: Fudan University (N=58), Shanghai Jiao Tong University (N=33), Sun Yat-sen University (N=22), Peking Union Medical College, Chinese Academy of Medical Sciences (N=15), and Memorial Sloan Kettering Cancer Center (N=14). Co-author analysis reveals that 60 institutions have published more than 7 papers. After excluding two unrelated institutions, a collaboration network diagram of 58 institutions is presented in **Figure 4**. As shown in the **Figure 4**, institutions belonging to the same cluster (denoted by the same color) are predominantly from the same country or belong to the same academic group. For example, Fudan University, Shanghai Jiao Tong University, and Shanghai Pancreas Institute, all located in Shanghai, collaborate closely and are grouped into the green cluster, while American institutions such as Harvard University, Mayo Clinic, and Northwestern University are grouped into the red cluster. The intricate network of connecting lines between clusters highlights the close collaboration between institutions from countries like China and the USA.

**Table 2:** The top 20 institution for publications.

Institution	Publications	Citations	Average citations
Fudan University	58	1191	21
Shanghai Jiao Tong University	33	626	19
Sun Yat-sen University	22	392	18
Chinese Academy of Medical Sciences & Peking Union Medical College	15	136	9
Memorial Sloan Kettering Cancer Center	14	518	37
Seoul National University	14	442	32
Johns Hopkins University	12	193	16
University of Amsterdam	9	145	16
Leiden University	8	295	37
Fox Chase Cancer Center	7	548	78

### 3.3 Analysis of publication authors

**Figure 5:**
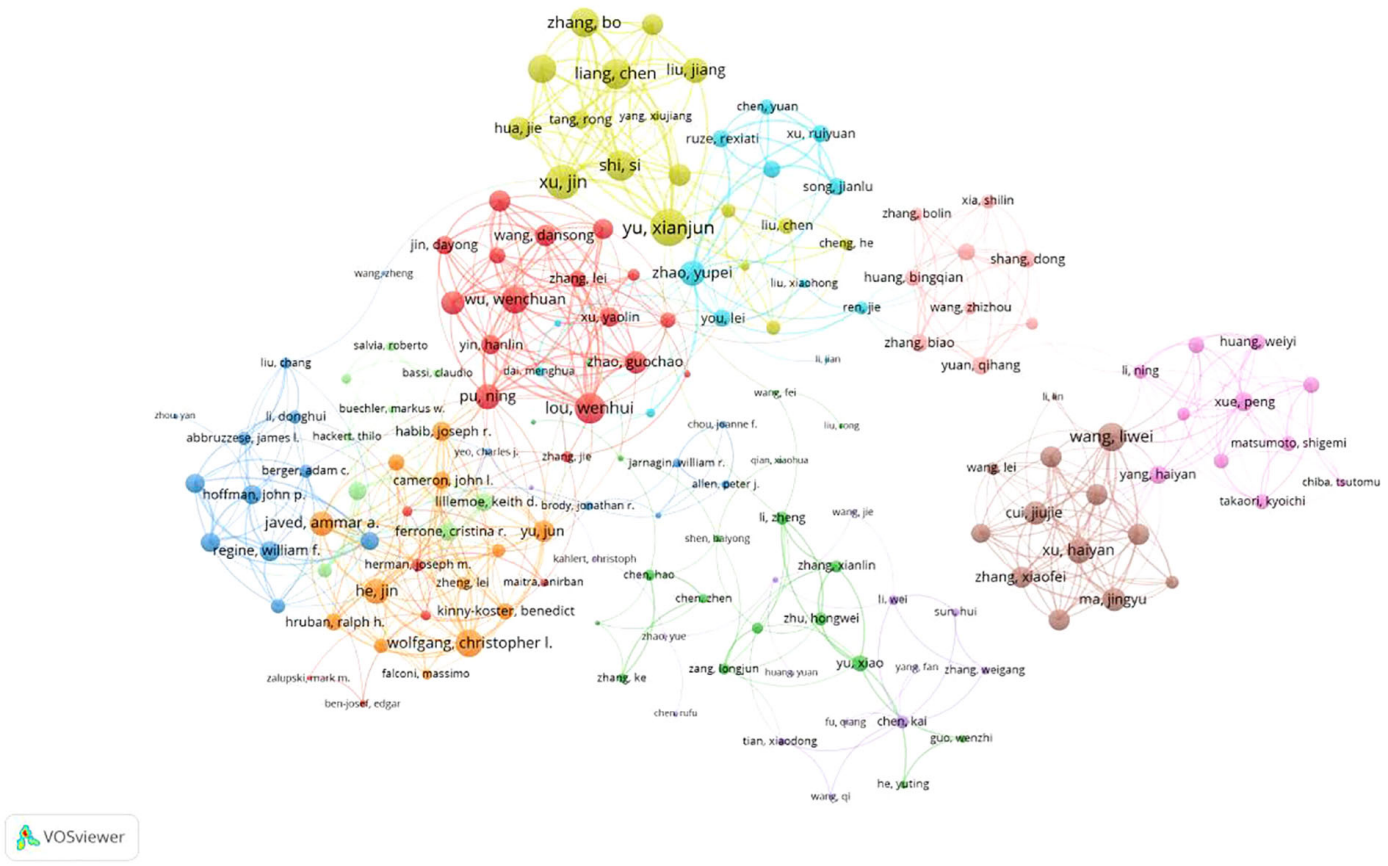
Network diagram of author collaborations for prognostic prediction model for pancreatic cancer.

A total of 6,716 authors have contributed to pancreatic cancer prognostic model research. The top 10 authors by publication volume and citation impact are listed in **Table 3**. The author with the most publications is Yu Xianjun from Fudan University (N=12), followed by Zhao Yupei from Peking Union Medical College Hospital (N=12). The author with the highest number of citations is Keith Lillemoe, followed by Martin Pichler and Markus Buechler. Additionally, several authors have high H-indices, including Yu Xianjun, Zhao Yupei, Lou Wenhui, and Jang, Jin-Young. Furthermore, according to Price’s Law, with the formula m = 0.749 N_max_ (where m represents the minimum number of publications for core authors and N_max_ is the number of publications by the most productive author), authors who have published 3 or more papers are considered core authors. we conducted a collaboration network analysis of authors who have published more than 3 papers (i.e., core authors) and found 10 different colored clusters of author groups in the collaboration network diagram (**Figure 5**). This demonstrates frequent communication among core authors, forming multiple academic groups represented by individuals such as Yu Xianjun, Zhao Yupei, Lou Wenhui, Christopher Wolfgang, and Markus Buechler. The dense internal connections within academic clusters reflect robust collaboration among core researchers, while sparse inter-cluster linkages highlight opportunities to strengthen cross-group partnerships.

**Table 3:** The top 10 authors for publications.

Author	H_index	Total publications	Total citations	Average citations
YU XIANJUN	8	12	383	32
ZHAO YUPEI	7	12	264	22
LOU WENHUI	7	9	176	20
JANG JIN-YOUNG	6	9	162	18
KEITH LILLEMOE	5	5	1396	279
MARTIN PICHLER	5	5	480	96
MARKUS BUECHLER	5	5	384	77
WILLIAM REGINE	5	5	365	73
STEFAN BOECK	5	5	297	59
SHI SI	5	5	201	40

### 3.4 Analysis of journals and cited journals

**Figure 6:**
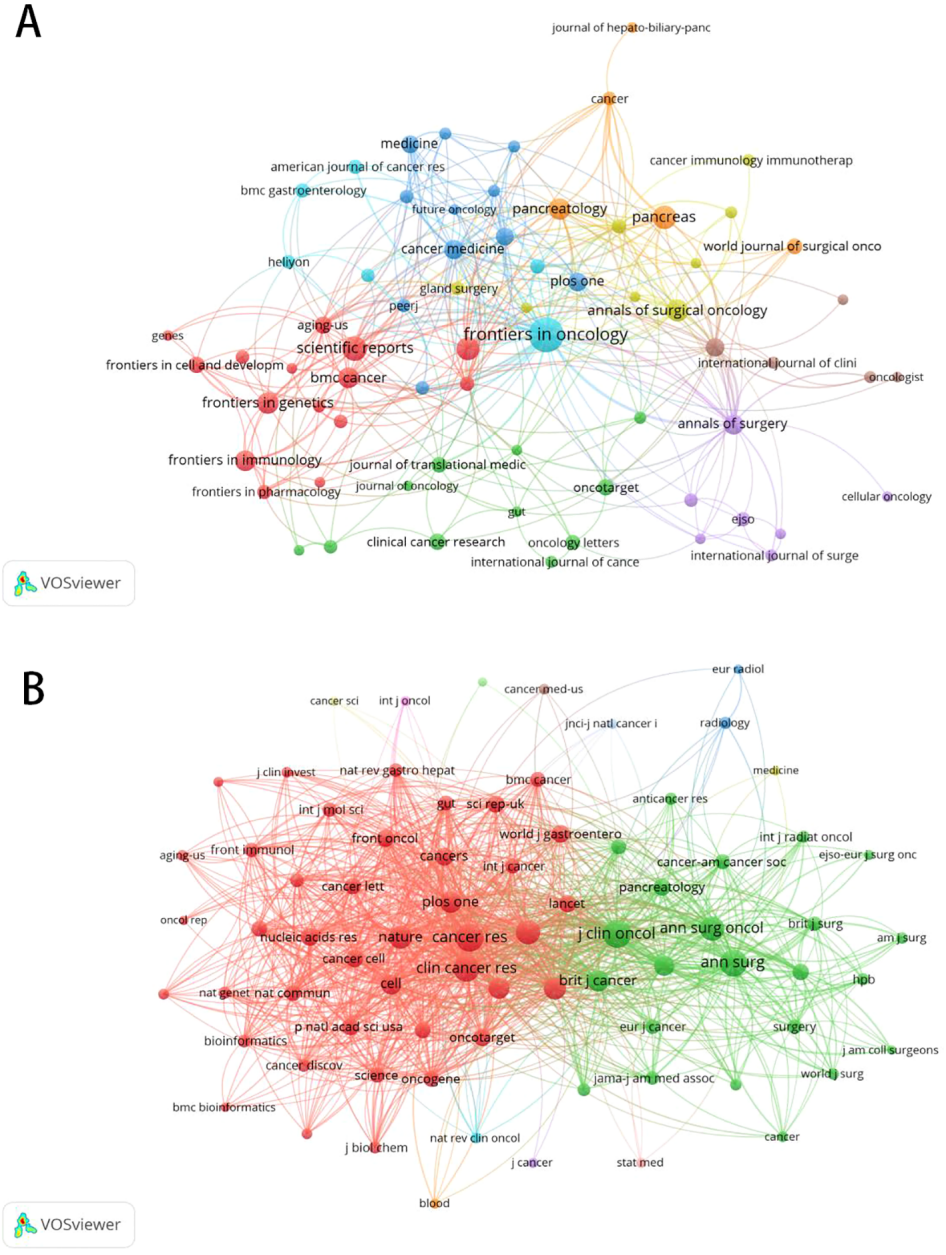
The citation and co-citation analysis of journal. **(A)** Network map of citation analysis of journals with more than five publications.**(B)**Network map of co-citation analysis of journals with more than one hundred citations.

**Figure 7:**
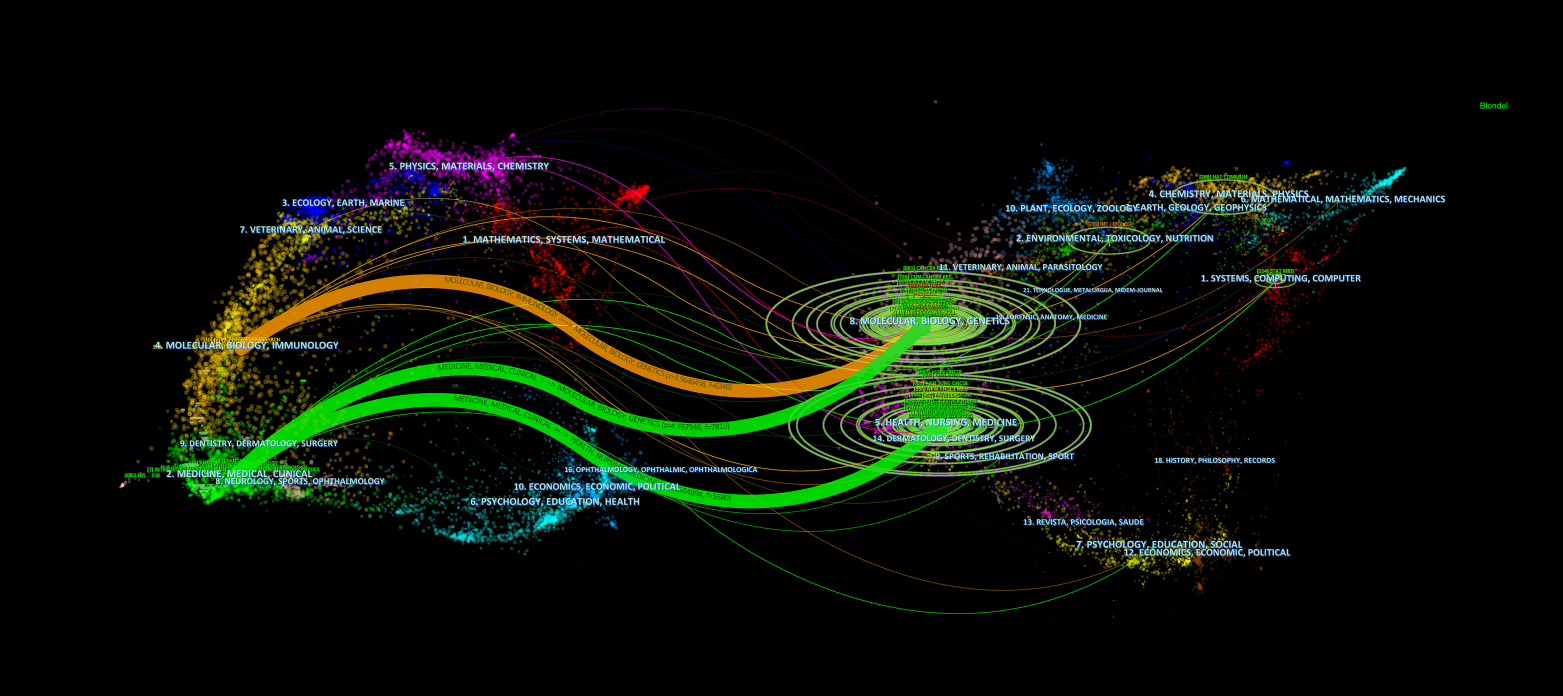
Double mapping overlay of journal research. The journals citing others are positioned on the left, while those being cited appear on the right.

A total of 303 journals have contributed to research on pancreatic cancer prognostic models. The citation analysis of these journals is shown in **Figure 6A**. **Table 4** shows the details of the top 10 journals with the largest number of published papers. *Frontiers in Oncology* leads in publication volume (52 papers), reflecting its role as a major open-access platform for oncology research, followed by *Pancreas* (N=23) and *Scientific Reports* (N=22). We found that 76 journals have been cited more than 100 times by these journals. The analysis of co-cited journals is shown in **Figure 6B**. Most of the cited journals belong to the fields of oncology and surgery, with a few in the fields of radiology, biochemistry, and others. The top 10 co-cited journals related to the published papers are included in the **Table 5**. The journal with the highest number of co-citations is *Cancer Research* (881 citations), followed by *Journal of Clinical Oncology* (852 citations) and *Annual of Surgery* (806 citations). **Figure 7** displays a two-graph overlay analysis of journal citation patterns. The journals citing others are positioned on the left, while those being cited appear on the right. The vertical dimension of each ellipse represents the number of publications per journal, and the horizontal dimension reflects the number of authors per journal. Three primary-colored routes show the relationships between the journals: the green path shows that journals in Molecular, Biology, Genetics, Health Sciences, Nursing, and General Medicine are consistently mentioned by medicine, medical, and clinical publications. Meanwhile, the orange route shows that Molecular, Biology, and Immunology cite publications are related to Molecular, Biology and Genetics.

**Table 4:** The top 10 sources for publications.

Journal	H_index	IF 2023	JCR 2023	Total publications	Total citations
FRONTIERS IN ONCOLOGY	10	3.5	2	52	321
PANCREAS	15	1.7	3	23	450
SCIENTIFIC REPORTS	9	3.8	1	22	321
CANCERS	9	4.5	1	21	358
ANNALS OF SURGICAL ONCOLOGY	11	3.4	1	18	641
PANCREATOLOGY	9	2.8	2	18	313
BMC CANCER	10	3.4	2	17	254
ANNALS OF SURGERY	13	7.5	1	15	781
JOURNAL OF GASTROINTESTINAL SURGERY	10	2.2	2	13	317
PLOS ONE	9	2.9	1	13	497

**Table 5:** The top 10 co-cited journals related to the published papers.

Journal	IF 2023	JCR 2023	Total citations
Cancer Research	11.7	1	881
Annals of Surgery	7.9	1	806
Clinical Cancer Research	10.4	1	736
Annals of Surgical Oncology	3.4	1	654
CA: A Cancer Journal for Clinicians	521.6	1	654
British Journal of Cancer	6.4	1	517
Cell	45.6	1	465
Cancers	4.5	1	368
Cancer Cell	48.8	1	341
Cancer Letters	9.1	1	300

### 3.5 References co-citation, clustering and bursts

**Table 6** lists the top 10 most cited publications on pancreatic cancer prognostic models. The most cited article is “Resected adenocarcinoma of the pancreas - 616 patients: Results, outcomes, and prognostic indicators” published in the JOURNAL OF GASTROINTESTINAL SURGERY (1230 citations), which included 616 patients from January 1984 to July 1999 and identified important prognostic factors.

**Table 6:** The top 10 most cited articles in studies.

Article title	Source	Institution	Cited	Year	DOI
Resected adenocarcinoma of the pancreas - 616 patients: Results, outcomes, and prognostic indicators	JOURNAL OF GASTROINTESTINAL SURGERY	Johns Hopkins University	1230	2000	10.1016/S1091-255X(00)80105-5
Increased neutrophil-lymphocyte ratio is a poor prognostic factor in patients with primary operable and inoperable pancreatic cancer	BRITISH JOURNAL OF CANCER	Medical University of Graz	420	2013	10.1038/bjc.2013.332
Prognostic nomogram for patients undergoing resection for adenocarcinoma of the pancreas	ANNALS OF SURGERY	Memorial Sloan Kettering Cancer Center	307	2004	10.1097/01.sla.0000133125.85489.07
Transcription analysis of human equilibrative nucleoside transporter-1 predicts survival in pancreas cancer patients treated with gemcitabine	CANCER RESEARCH	University of Pisa	294	2006	10.1158/0008-5472.CAN-05-4203
Postresection CA 19-9 Predicts Overall Survival in Patients with Pancreatic Cancer Treated with Adjuvant Chemoradiation: A Prospective Validation by RTOG 9704	JOURNAL OF CLINICAL ONCOLOGY	Jefferson University	282	2008	10.1200/JCO.2008.18.6288
Prognostic factors in resected pancreatic adenocarcinoma: Analysis of actual 5-year survivors	JOURNAL OF THE AMERICAN COLLEGE OF SURGEONS	University of Toronto	265	2004	10.1016/j.jamcollsurg.2004.01.008
ACUTE-PHASE PROTEIN RESPONSE AND SURVIVAL DURATION OF PATIENTS WITH PANCREATIC-CANCER	CANCER	University of Edinburg	252	1995	10.1002/1097-0142(19950415)75:8<2077:AID-CNCR2820750808>3.0.CO;2-9
Identification of MicroRNA-21 as a Biomarker for Chemoresistance and Clinical Outcome Following Adjuvant Therapy in Resectable Pancreatic Cancer	PLOS ONE	National Institutes of Health (NIH) - USA	244	2010	10.1371/journal.pone.0010630
Comparison of the prognostic values of various inflammation-based factors in patients with pancreatic cancer	MEDICAL ONCOLOGY	Sun Yat Sen University	190	2012	10.1007/s12032-012-0226-8
The Systemic-immune-inflammation Index Independently Predicts Survival and Recurrence in Resectable Pancreatic Cancer and its Prognostic Value Depends on Bilirubin Levels A Retrospective Multicenter Cohort Study	ANNALS OF SURGERY	Erasmus University Rotterdam	182	2019	10.1097/SLA.0000000000002660

Co-citation analysis reveals frequently referenced foundational works. The top three co-cited references are: 1) Siegel et al. (2023, CA: A Cancer Journal for Clinicians; 105 citations); 2) Conroy et al. (2011, New England Journal of Medicine; 97 citations); 3) Rahib et al. (2014, Cancer Research; 97 citations). Notably, two focus on pancreatic cancer epidemiology, underscoring its influence in prognostic model development.

**Figure 8:**
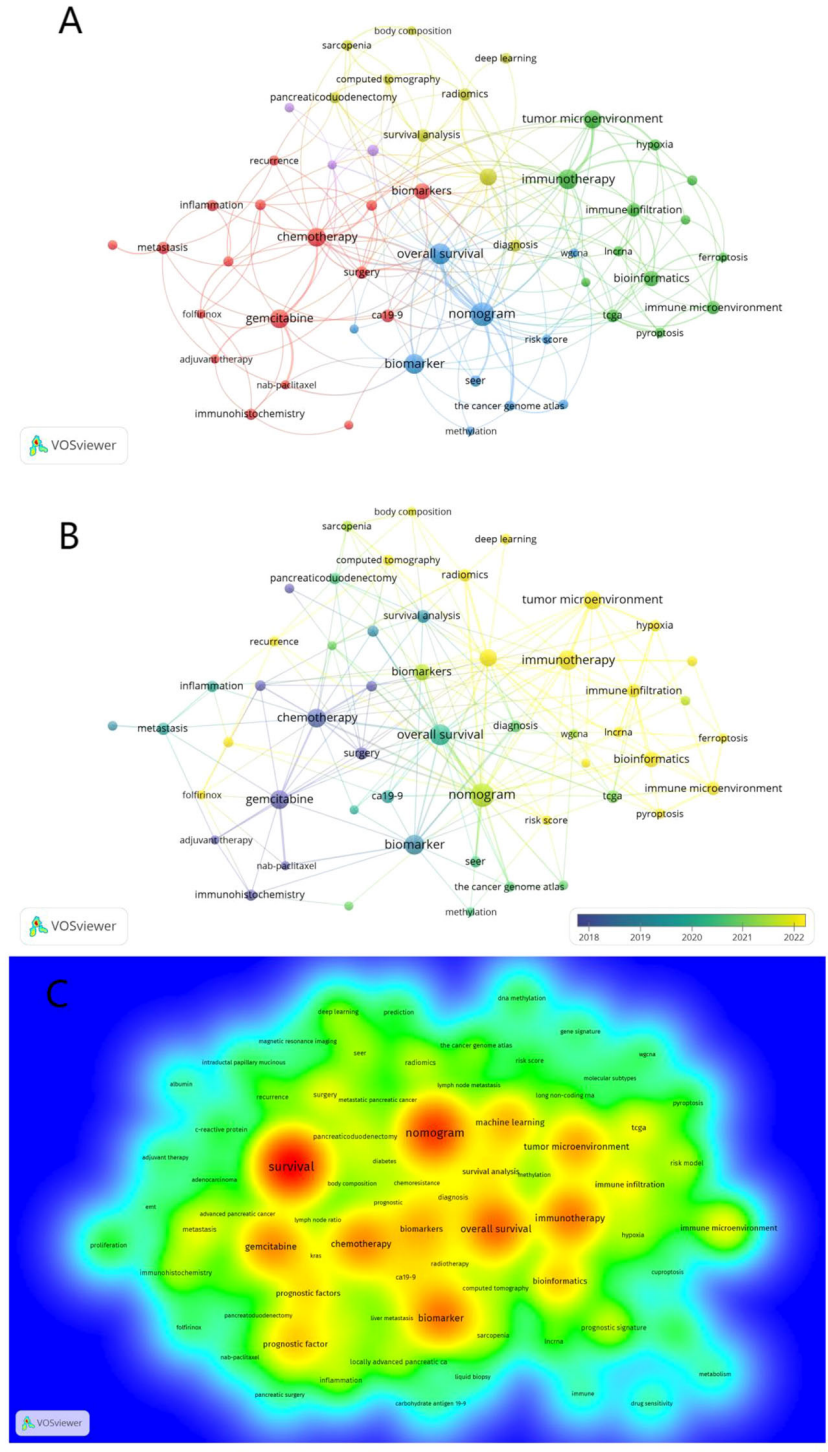
Co-occurrence analysis of keywords. **(A)** Co-occurrence clustering map of keywords. **(B)** The keyword frequency-time map. The size of the circles is correlated with the frequency of keyword occurrences; blue represents earlier keyword occurrences, yellow represents later keyword occurrences **(C)** Density visualization of keywords.

**Figure 9:**
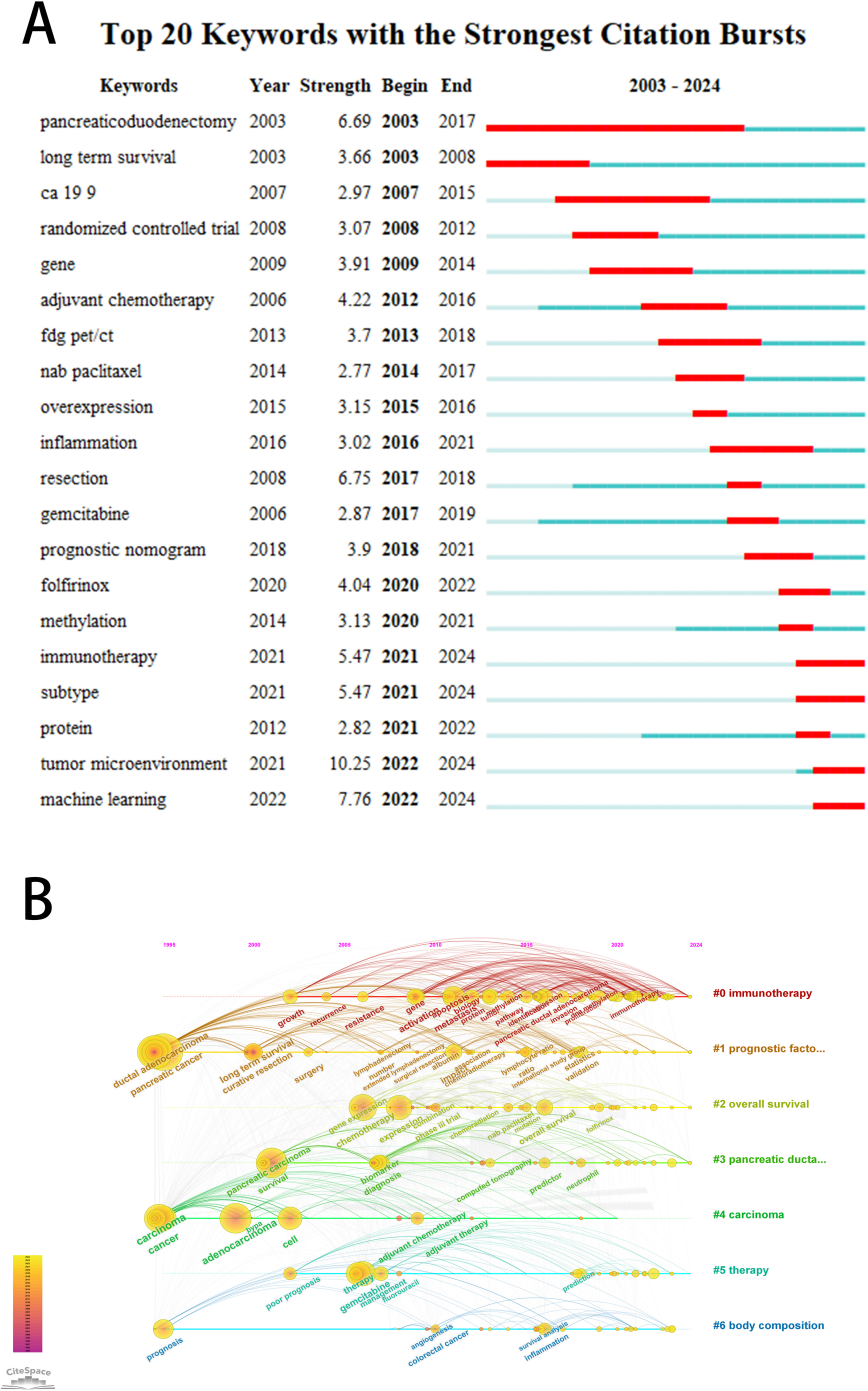
**(A)** The top 20 keywords with the strongest citation bursts. The bar graph on the right shows the position of the reference burst duration (red part) of each keyword in the whole time span (2003-2024). **(B)** Keyword clustering time diagram. Different colored paths and node groups represent different clustering of research topics.

The purposes of keyword co-occurrence analysis and burst analysis are to study the relationships among keywords in a set of publications, identify hot topics, explore the changes of these topics over time, and assist scholars in better understanding current scientific concerns. Using VOSviewer for clustering, time and density co-occurrence analysis of “author keywords” (**Figures 8A–C**), a total of 1669 keywords were identified. Based on the clustering analysis results, these keywords can be divided into four clusters: 1)Chemotherapy Regimens and Biochemical Indicators for Pancreatic Cancer, including keywords such as gemcitabine, inflammation, and CA19-9. 2)Imaging and Artificial Intelligence in Pancreatic Cancer, with keywords like CT, radiomics, and machine learning. 3)Tumor Microenvironment and Genetics of Pancreatic Cancer, encompassing keywords such as immune microenvironment, ferroptosis, hypoxia, lncRNA, and TCGA. 4) other type of keywords, such as overall survival and nomogram. Using Citespace for keyword burst detection (**Figure 9A**), the results are similar to those of VOSviewer. Keywords such as CA19-9 and pancreaticoduodenectomy emerged earlier, while keywords like PET/CT, gene, and inflammation appeared frequently in the mid-period. Keywords with rising prominence (2021–2024) reflect a strategic shift toward integrative research themes, notably tumor microenvironment dynamics, machine learning-driven prognostic modeling, and protein-based biomarker discovery. **Figure 9B** illustrates the time axis analysis of keyword clustering. The size of each sphere is proportional to the keyword frequency; The connection between spheres indicates keyword co-occurrence. The color of the sphere represents the time span of the occurrence of keywords. Keywords in the same group are arranged horizontally. The earliest node appears at the top and advances to the right with time. This configuration can visualize the number and time distribution of keywords, highlighting their importance and duration. The number of keywords in each group can be obtained from the figure. The more keywords there are, the more important the group field is. In addition, the keywords were divided into six different groups, including #0 immunotherapy, #1 prognostic factors, #2 overall survival, #3 pancreatic ductal adenocarcinoma, #4 carcinoma, #5 therapy, #6 body composition. These analyses provide insights into the evolution of research interests in the field of pancreatic cancer, highlighting the emergence of new topics and the persistence of classic research areas.

## 4 Discussion

To the best of our knowledge, this is the first bibliometric analysis focused on pancreatic cancer prognostic model research. The study reveals a continuous upward trend in the annual number of publications in this field. The significant increase in publications and citations in recent years reflects clinicians’ growing attention toward pancreatic cancer prognostic model research. The year 2020 marks an important milestone in this field, yielding both the highest number of citations (1652) and the highest H-index (H =22). The relatively lower citation count and H-index observed in the past four years may be attributed to the proximity of the data collection cutoff date.

By analyzing the countries, institutions, authors, and journals in this field, we found that the United States has a significant leadership position, with the most extensive international cooperation network and the highest number of citations. China ranks first in publication volume, and Chinese institutions occupy four of the top five positions for productivity. Among these, Fudan University is the most productive and influential research institution. Yu Xianjun from Fudan University is the most influential scholar in this field. However, despite the substantial research manpower and funding invested by China in this field, and possessing the most influential institutions and scholars, the average number of citations per paper is relatively low. This suggests that there is considerable variation in the quality of academic papers produced in China, which may be a side effect of the country’s large population size on research, or it may be due to the limited collaboration between most Chinese researchers and those from other countries/regions and institutions. Therefore, for China, it is urgent to strengthen international cooperation and exchanges. Researchers can integrate global resources and expand the international influence of research results by integrating into the international top research alliance, building joint laboratories, and cooperating to apply for major projects. Deepen substantive exchanges among scholars: the government can break through the communication barriers by increasing overseas research opportunities and encouraging Chinese and foreign scholars to jointly publish papers.

*Frontiers in Oncology* is the journal with the highest number of publications in this field, possibly because it is a multidisciplinary journal covering various aspects of cancer and has a large total number of publications. *Annals of Surgery* is a recognized top journal in surgery and has the highest number of citations in this field. Most of the journals that rank high in terms of publication volume and citations belong to the JCR Q1/Q2 quartile, indicating their significant academic influence. Research on prognostic prediction models for pancreatic cancer is mostly published in surgical and oncological journals and has been heavily cited. Therefore, researchers can refer to this study in their future work and strive to publish their papers in high-level journals in the fields of surgery and oncology.

The field of prognostic prediction models for pancreatic cancer has gathered many outstanding researchers (**Table 3**). An analysis of their published articles shows that Yu Xianjun and Zhao Yupei from China have published the most papers. They are committed to exploring novel prognostic factors in immunology, genomics, and pathology (12). Jang Jin-Young from Seoul National University focuses on the application of imaging and machine learning in the prognosis prediction of pancreatic cancer (13, 14). Although European and American leaders in pancreatic surgery, such as Markus Buechler and Keith Lillemoe, have published relatively few papers, their work is foundational in this field (15). These outstanding authors focus on various fields, and subsequent researchers can seek international exchange and cooperation based on the above research results. It is noteworthy that outstanding scholars in Europe and America generally entered this research field earlier, which may be one of the reasons for their higher citation rates. In contrast, although Chinese scholars have published a considerable number of papers, they entered the field relatively later, resulting in relatively lower citation rates.

An analysis of the top ten most cited articles reveals that, using a 10-year cutoff, there is only one article from 2014-2024, seven from 2004-2014, and two from 1995-2004. There are relatively few highly cited research articles in the past decade (2014–2024), which may be related to the shorter time span and insufficient accumulation of citations. However, this phenomenon may also reveal that the research and innovation of pancreatic cancer has encountered a huge “bottleneck”. This bottleneck is mainly reflected in two aspects: 1) pancreatic cancer itself is highly heterogeneous, lacking effective biomarkers like other cancers, and the progress of treatment methods is limited in the past decade, which leads to the reduction of the clinical practical value of the new prediction model. 2) High quality evidence is scarce. In the past decade, many studies only use new algorithms such as machine learning to analyze old data, lacking substantive breakthroughs and repeatability. Future breakthrough research should focus on the prediction model associated with treatment decision-making, and establish large-scale, forward-looking, multi-center international cooperation. The model based on this will have higher credibility and influence.

From the keyword co-occurrence and cluster analysis, firstly, the research direction of prognostic predictors for pancreatic cancer has gradually shifted from patient clinical risk factors, biomarkers and pathological features to the tumor microenvironment, bioinformatics, imaging, etc. Secondly, keywords also include terms related to statistical and modeling methods, such as nomograms and randomized controlled trials. In addition, keywords predicting clinical outcomes such as survival, long-term survival, and lymph node metastasis also appear in the co-occurrence analysis. Therefore, based on the results of keyword co-occurrence analysis, we divide the prognostic prediction models for pancreatic cancer into three parts: predictive factors, statistical modeling, and clinical outcomes, which will be introduced separately here.

### 4.1 Predictive factors of prognostic prediction models for pancreatic cancer

The research hotspots of predictive factors for prognostic prediction in pancreatic cancer have changed significantly over time. Therefore, we will discuss the research hotspots and keywords of predictive factors for prognostic prediction in pancreatic cancer in roughly chronological order.

In the early days, the prognostic models for pancreatic cancer primarily incorporated predictive factors focused on the patient’s basic clinical information. A landmark study (15), which is the most cited in this field, identified three key independent prognostic factors: tumor characteristics, R0 resection status, and chemoradiotherapy status. These findings laid the foundation for subsequent research. However, these indicators also have certain limitations. For example, indicators based on basic clinical information lack specificity, and pancreatic cancer is a type of tumor with high heterogeneity, posing challenges for the clinical application of such prognostic models. This prompts researchers to continue seeking new prognostic indicators for pancreatic cancer.

Inflammation-based biochemical indicators have been a persistent research hotspot for predicting the prognosis of pancreatic cancer, showing explosive growth in the early to mid-stages. Inflammation has been confirmed to play a key role in inducing malignant transformation of pancreatic epithelial cells, promoting pancreatic cancer metastasis, and affecting the efficacy of chemotherapy (16). Moreover, inflammation indicators are simple to test, easy to operate, low in cost, and capable of dynamically monitoring disease progression, making them widely applicable in clinical practice. A study (17) compared the prognostic values of commonly used inflammatory factors and found that the neutrophil/lymphocyte ratio (NLR) (HR, 2.537; 95% CI, 1.313–4.902) outperformed modified Glasgow Prognostic Score (mGPS), Prognostic Index (PI), platelet/lymphocyte ratio (PLR), and Prognostic Nutritional Index (PNI) in predicting the prognosis of pancreatic cancer patients, serving as an independent predictor of survival. This is also consistent with the conclusions of a meta-analysis (18). Interestingly, however, in another large-scale meta-analysis (19), Wu et al. considered mGPS as a promising tool for predicting pancreatic cancer. Therefore, how to screen an increasing number of inflammatory biochemical markers and further improve the predictive ability of prediction models is a complex issue for subsequent researchers. Future studies should pay attention to it.

Histologically, pancreatic cancer is a dense matrix composed of various cellular and non-cellular components, known as the tumor microenvironment (TME) (20). In recent years, research on prognostic prediction models for pancreatic cancer has entered a period of rapid development, primarily due to the explosive growth in research on the tumor microenvironment of pancreatic cancer. According to keyword co-occurrence analysis, prognostic prediction models related to the tumor microenvironment of pancreatic cancer roughly revolve around the following directions: 1) Immune microenvironment, which includes immune cells such as tumor-associated macrophages and neutrophils, immune checkpoint molecules like PD-1, and tumor antigens such as CEA. Keywords include immune microenvironment, pyroptosis, etc. In a relatively new study (21), researchers found that S100A2 is a prognostic biomarker that participates in the immune microenvironment of pancreatic cancer and predicts the response to immunotherapy. 2) Fibrosis and stromal microenvironment, with keywords such as cancer-associated fibroblasts (CAFs) and extracellular matrix (ECM). Studies using single-cell RNA and protein analysis have identified tumor-stromal interactions in the TME of pancreatic cancer that determine the molecular characteristics and clinical prognosis of tumor cells (22). 3) Metabolic microenvironment, with keywords including hypoxia and ferroptosis. Research on hypoxia emerged earlier and has been ongoing. A study (23) demonstrated that the expression of PDGFA, bFGF, and HIF-1α significantly impacts prognosis. A more recent highly cited article (24) focused on the metabolic process of HIF1-α and discovered that the transcription factor protein TCF7L2 can serve as a new prognostic biomarker. Research on ferroptosis is still limited. Tang et al. (25) developed an accurate prognostic model based on ferroptosis-related lncRNA expression patterns, providing a new predictive approach for the prognosis of pancreatic cancer. 4) Epigenetic regulation, with keywords including methylation and lncRNA. In a study (26), regulators involved in m6A RNA methylation were found to be related to the development of pancreatic cancer. The m6Ascore signature is proposed as an indicator of TME status, aiding in predicting the prognosis of pancreatic cancer patients.

Another promising direction is artificial intelligence. Artificial intelligence is a branch of computer science that uses computers to mimic human brain processes and intelligent behavior (27). With the development of subfields of artificial intelligence such as machine learning and deep learning technologies, artificial intelligence is now playing a role in various aspects of prognostic prediction (28), with radiomics being one of the most notable areas of focus. Radiomics can mine quantitative image features in a high-throughput manner, extract data, and improve the accuracy of diagnosis, prognosis, and prediction (29). Radiomics is most widely applied in CT (30–32). It is worth noting that Imamate (33) combined CT imaging with genomics, proving that radiogenomics can predict p53 mutations in patients with pancreatic cancer, thus predicting the prognosis of patients with PDAC. Furthermore, MRI imaging technology has rapidly developed in recent years, and research on MRI-based radiomic prognostic prediction is gradually increasing (34, 35), although the overall number remains limited. This may be attributed to the following reasons: 1) Compared to CT imaging, MRI technology is complex and susceptible to artifacts, making it difficult to standardize MRI images from different devices and scanning conditions, which affects the extraction and reproducibility of radiomic features (36, 37). 2) Due to the relatively low incidence of pancreatic cancer and limited MRI scanning (long scan times, high costs), it is challenging to obtain large-scale, high-quality MRI image datasets. 3) The “black box” problem posed by machine learning is a common challenge in radiomics. The lack of interpretability of prognosis-related features obtained through radiomics reduces the credibility of the model to some extent (38). The research situation for PET/CT radiomics is similar to that of MRI (39, 40). Nevertheless, the future of radiomics is promising, with researchers striving to further develop radiomics by establishing standardization protocols, sharing databases, and conducting multimodal radiomic studies (41, 42).

In summary, clinical data such as tumor size, location, patient age, TNM stage, negative margin resection, surgical procedure, radiotherapy/chemotherapy, CA19-9 and lymph node or distant metastasis remain the most studied, widely used, and recognized indicators for pancreatic cancer. Considering the obvious limitations of these indicators, researchers should use more advanced prediction models in the future. At present, the state-of-the-art method of prediction model for pancreatic cancer prognosis should include: 1) Integrate multi-dimensional data of clinical, imaging (such as ct/mri) and molecular biology. 2) Advanced machine learning or deep learning algorithms. 3) Strict multi center, prospective external verification. 4) It has good explainability and can be easily deployed as a clinical decision support tool.

### 4.2 Statistical methods for prognostic prediction models of pancreatic cancer

This article systematically analyzes the statistical modeling methods employed in the included studies, revealing several noteworthy research characteristics and potential limitations. Firstly, it is evident that most models are developed based on retrospective data. Although prospective study designs are more suitable for developing prognostic models, given the constraints of research conditions in the medical field, researchers often must sacrifice some data quality in exchange for the advantages of long-term follow-up and cost savings associated with retrospective data.

In model validation of pancreatic cancer prognosis prediction models, several challenges exist. First, external validation is insufficient - a meta-analysis showed only 69.2% of models underwent validation, with 52.9% using external patient cohorts. Among externally validated models, 66.7% used independent datasets, while only 11.1% were validated by different authors (43). Second, for artificial intelligence-based models, small sample sizes combined with numerous parameters often lead to overfitting, an issue frequently overlooked. Future research should emphasize model validation, with multi-center and independent external validation planned from the study design phase. When feasible, validation through prospective RCTs or cohort studies is recommended. For complex machine learning models, researchers can employ methods like SHAP to explain model predictions and enhance clinical trust.

Nomograms are the only modeling method that appears in the keyword analysis, indicating their popularity among researchers. Kaplan-Meier curves and multivariate Cox proportional hazards regression are also commonly used modeling methods. Furthermore, with advancements in artificial intelligence, machine learning methods such as Bayesian models and artificial neural networks (ANNs) are gradually emerging as new modeling approaches, providing new options for future researchers.

### 4.3 Selection of clinical outcomes of prognostic models for pancreatic cancer

In studies on prediction models for pancreatic cancer, the selection of clinical outcomes is a crucial issue. Keyword analysis reveals that overall survival (OS), long-term survival, and disease-free survival (DFS) are frequent terms, indicating that researchers tend to use survival-related indicators as the primary clinical outcomes in prediction models. Additionally, treatment response, another high-frequency clinical outcome indicator, has also received widespread attention from researchers. For example, Martinelli et al. (44) selected adjuvant treatment response as the predictive outcome and successfully demonstrated a predictive association between the loss of GATA6 expression and poor adjuvant treatment response. Throughout the development of prediction models for pancreatic cancer, early studies primarily focused on survival predictions, a choice that is justifiable given the universal applicability of survival as a clinical outcome, facilitating comparison and validation among different studies. As research deepens, indicators such as recurrence rate, R0 resection rate, pancreatic fistula, lymph node metastasis, and liver metastasis have been gradually incorporated into clinical outcome studies. Particularly noteworthy is the pioneering study (45), which explored the association between hospital inpatient capacity and R0 resection rate in pancreatoduodenectomy from the hospital level and ultimately demonstrated that low inpatient capacity is a significant factor influencing positive R0 resection margins. Although the main advantage of this study may lie in its sample size (12,101 patients), subsequent researchers can also draw inspiration from its approach to explore potential associations between non-traditional influencing factors and non-traditional clinical outcomes.

Bibliometric analysis, as an important research method, helps systematically organize the development trajectory and research foci of prognostic prediction models for pancreatic cancer. However, it must be acknowledged that this research method also has inevitable limitations. This study has several limitations. First, using only the WoSCC database may not provide comprehensive coverage compared to including other databases like Scopus and PubMed. Future research should consider multi-database search strategies with standardized deduplication. Second, the literature retrieval deadline of October 2024 limits capturing recent trends. A dynamic bibliometric analysis approach with regular updates could address this limitation. Third, the inherent limitations of bibliometric software may result in overlooking certain scholarly contributions. Using multiple analysis tools (e.g., VOSviewer and CiteSpace) for cross-validation could enhance result reliability. Finally, including only “article” type publications may introduce bias, though the impact is likely minimal. Future studies could analyze different publication types separately.

Research on prognostic prediction models for pancreatic cancer is currently in a steady stage of development. Existing studies have primarily focused on biochemistry, tumor microenvironment analysis, bioinformatics, imaging, and artificial intelligence. Looking ahead, several specific research directions are critical for advancing the field. First, multicenter validations are needed to ensure the robustness and generalizability of these models across diverse patient populations and clinical settings. Second, efforts toward model standardization will facilitate consistent methodology and allow for effective comparisons between studies. Additionally, Future models should use the ability of multimodal data integration to combine genomic, imaging and clinical data to build a more comprehensive and accurate prediction system. This bibliometric study not only elucidates the current state of prognostic prediction models for pancreatic cancer but also highlights key future avenues—such as multicenter validation, model standardization, and the incorporation of emerging multi-dimensional data—that can help researchers uncover new hotspots, frontiers, and innovative research directions in the discipline.

## Data Availability

The original contributions presented in the study are included in the article/supplementary material. Further inquiries can be directed to the corresponding authors.
